# A Survey of Copy Number Variation in the Porcine Genome Detected From Whole-Genome Sequence

**DOI:** 10.3389/fgene.2019.00737

**Published:** 2019-08-16

**Authors:** Brittney N. Keel, Dan J. Nonneman, Amanda K. Lindholm-Perry, William T. Oliver, Gary A. Rohrer

**Affiliations:** USDA, ARS, U.S. Meat Animal Research Center, Clay Center, NE, United States

**Keywords:** copy number variation, swine, whole-genome sequence, read depth, olfactory receptor

## Abstract

Copy number variations (CNVs) are gains and losses of large regions of genomic sequence between individuals of a species. Although CNVs have been associated with various phenotypic traits in humans and other species, the extent to which CNVs impact phenotypic variation remains unclear. In swine, as well as many other species, relatively little is understood about the frequency of CNV in the genome, sizes, locations, and other chromosomal properties. In this work, we identified and characterized CNV by utilizing whole-genome sequence from 240 members of an intensely phenotyped experimental swine herd at the U.S. Meat Animal Research Center (USMARC). These animals included all 24 of the purebred founding boars (12 Duroc and 12 Landrace), 48 of the founding Yorkshire-Landrace composite sows, 109 composite animals from generations 4 through 9, 29 composite animals from generation 15, and 30 purebred industry boars (15 Landrace and 15 Yorkshire) used as sires in generations 10 through 15. Using a combination of split reads, paired-end mapping, and read depth approaches, we identified a total of 3,538 copy number variable regions (CNVRs), including 1,820 novel CNVRs not reported in previous studies. The CNVRs covered 0.94% of the porcine genome and overlapped 1,401 genes. Gene ontology analysis identified that CNV-overlapped genes were enriched for functions related to organism development. Additionally, CNVRs overlapped with many known quantitative trait loci (QTL). In particular, analysis of QTL previously identified in the USMARC herd showed that CNVRs were most overlapped with reproductive traits, such as age of puberty and ovulation rate, and CNVRs were significantly enriched for reproductive QTL.

## Introduction

One of the important challenges in post-genomic biology is relating observed phenotypic variation to the underlying genotypic variation. Genome-wide association studies (GWAS) have made thousands of connections between single-nucleotide polymorphisms (SNPs) and phenotypes, implicating regions of the genome that may play a causal role in a variety of complex traits. Despite their success in identifying associated variants, association studies account for only a small percentage of the total heritability (Maher, 2008). Hence, determining other types of variation that may make a substantial contribution to variation in complex traits is a meaningful goal.

Copy number variations (CNVs) are gains and losses of large regions of genomic sequence between individuals of a species, ranging from kilobases to megabases in length (Feuk et al., 2006). It is hypothesized that CNVs represent a significant source of genetic variation, as they have been shown to cover approximately 7% of the mouse genome (Locke et al., 2015), 12% of the human genome (Redon et al., 2006), and 7% of the cattle genome (Keel et al., 2016a). Significant overlap between protein-coding genes and CNV has been reported in a number of species, including human (Bailey et al., 2009), mouse (Locke et al., 2015), cattle (Keel et al., 2016b), and pig (Paudel et al., 2013). Conrad et al. (2010) found that 40% of validated CNV overlapped with at least one gene. In addition, CNVs appear to influence gene expression levels (Stranger et al., 2007; Henrichsen et al., 2009).

In humans and rodents, CNVs have been well studied and linked to various phenotypic traits and diseases (Cook and Scherer, 2008; Almal and Padh, 2012; Girirajan et al., 2013). Initial CNV studies have been performed in a number of domesticated animals: dog (Nicholas et al., 2011; Alvarez and Akey, 2012; Berglund et al., 2012), cattle (Fadista et al., 2010; Liu et al., 2010; Hou et al., 2011; Stothard et al., 2011; Zhan et al., 2011; Bickhart et al., 2012; Hou et al., 2012a; Hou et al., 2012b; Jiang et al., 2012; Choi et al., 2013; Wu et al., 2015; Keel et al., 2016a), sheep (Fontanesi et al., 2011; Liu et al., 2013), chicken (Crooijmans et al., 2013; Yi et al., 2014), and goat (Fontanesi et al., 2010).

Swine CNVs have been reported using a variety of array-based platforms, including comparative genomic hybridization arrays (Fadista et al., 2008; Li et al., 2012; Wang et al., 2014; Wang J. et al., 2015), the Illumina PorcineSNP60 BeadChip (Ramayo-Caldas et al., 2010; Chen et al., 2012; Wang et al., 2012; Wang L. et al., 2013; Schiavo et al., 2014; Wiedmann et al., 2015; Xie et al., 2016; Zhou et al., 2016; Hay et al., 2017), and the Illumina Infinium II Multisample SNP assay (Wang J. et al., 2013; Long et al., 2016). These approaches are known to suffer some drawbacks, including limited coverage of the genome due to low probe density, low resolution, and hybridization noise (Zhao et al., 2013). Ongoing developments and cost decreases in next-generation sequencing (NGS) technology have led to an increased popularity of sequence-based CNV detection. To date, a limited number of studies have utilized NGS data to identify CNV in the porcine genome.

The number and size ranges of CNV detected in previous swine studies utilizing NGS vary dramatically. These discrepancies may be artifact of differences in many aspects of the study, including sequence coverage, sample size, breed, and CNV detection algorithm. In swine, as well as many other species, relatively little is known about the properties of CNV, including their frequency in the genome, sizes, locations, and chromosomal properties. Of all the topics related to CNV, knowledge of their functional impact is the most limited. Despite the wide range of number and size of CNV reported between previous swine NGS studies, the results from functional enrichment analysis of CNV are quite consistent. Gene ontology (GO) terms related to sensory perception (Paudel et al., 2013; Jiang et al., 2014; Paudel et al., 2015), response to stimuli (Paudel et al., 2013; Jiang et al., 2014), immunity (Jiang et al., 2014; Paudel et al., 2015), and olfactory receptor (OR) activity (Paudel et al., 2015; Revilla et al., 2017) were the most significant in these studies. The same GO terms have been identified in CNV studies in humans and cattle. ORs, which are G-protein-coupled receptors involved in signal transduction, play a role in all the GO terms listed above. The results from previous studies suggest that CNVs may play a role in olfactory ability and sensitivity, which may be related to economically relevant traits in swine including feeding behavior (Connor et al., 2018) and reproduction (Baum and Cherry, 2015).

The CNVs reported in the aforementioned studies represent several diverse pig breeds and wild boars from different regions of the world. Very few animals in these studies (only 37 of 353) represent commercial swine germplasm, which, through domestication, has been shaped by selection for docility and lean meat production. Additionally, previous CNV studies in swine have been conducted using the Sscrofa 9.2 and 10.2 genome builds. The purpose of this study is to identify and characterize CNV regions detected from whole-genome sequence of 240 members of an experimental swine herd at the U.S. Meat Animal Research Center (USMARC), a resource representative of commercial swine germplasm, utilizing the newly released, high-quality Sscrofa 11.1 genome assembly.

## Materials and Methods

The DNA samples sequenced for this study were extracted from semen, blood, and tail tissue archived under standard operating procedures for the U.S. Meat Animal Research Center tissue repository. The research did not involve experimentation on animals requiring IACUC approval.

### Sequencing and Data Acquisition

CNVs were detected from whole-genome sequence of 240 members of an experimental swine herd. This composite population, developed at USMARC, began in 2001 by mating mixed Landrace-Yorkshire sows with 24 purebred founding boars—12 Landrace and 12 Duroc. To produce the second generation, Landrace-sired animals were mated to Duroc-sired animals. Subsequent generations were produced by selecting 1 male and 10 females produced by each founding boar and then randomly mating them, avoiding full-sib and half-sib pairings (Lindholm-Perry et al., 2009). Industry sires were then introduced in generation 10 and used in subsequent generations. This study utilizes whole-genome sequence from all 24 founding boars, 48 of the founding sows, and 109 animals from generations 4 through 9, 29 animals from generation 15, and 30 purebred industry boars (15 Landrace and 15 Yorkshire) used as sires in generations 10 through 15.

DNA extraction and library preparation have been previously described in Keel et al. (2017) and Keel et al. (2018) for the 72 founding animals and the remaining 168 animals, respectively. Libraries were paired-end sequenced (150 bp read length) on an Illumina NextSeq500 (Illumina, San Diego, CA, USA) at USMARC. Bases of the paired-end reads for all sequenced genomes were identified with the Illumina BaseCaller, and FASTQ files were produced for downstream analysis of the sequence data. Sequence data are available for download from the National Center for Biotechnology Information (NCBI) Sequence Read Archive (SRA) BioProjects PRJNA343658, PRJNA414091, and PRJNA482384.

### Sequence Data Processing

The Trimmomatic software (Version 0.35; Bolger et al., 2014) was used to trim Illumina adaptor sequences and low-quality bases from the reads. The quality cutoff was a PHRED33 score of >15. Reads containing any portion with an average PHRED33 score <15 spanning at least 4 bp were removed. The remaining reads were mapped to the Sscrofa 11.1 genome assembly using Burrows-Wheeler Alignment (BWA, Version 0.7.12; Li and Durbin, 2009) with the default parameters.

### CNV Detection and Defining CNVRs

A combination of the CNVnator (Version 0.3.2; Abyzov et al., 2011) and LUMPY (Version 0.4.13; Layer et al., 2014) software was used to identify putative CNV in the genome sequence of the 240 pigs. LUMPY is a probabilistic CNV discovery framework that integrates multiple detection signals, including split reads and paired-end mapping, while the CNVnator is a read depth method that uses a mean-shift-based approach to call CNV based on the depth of sequencing.

CNVs were first called for each sample using the CNVnator. The program was run using a window size (bin size) of 1 kb, and all other parameters were set to the default. Next, CNVs were detected using LUMPY with default parameters. CNV breakpoints from the CNVnator output were passed as input into LUMPY using the –bedpe option.

In an attempt to reduce the number of false positives, CNVs were also called using the cn.MOPS algorithm (Version 1.24.0; Klambauer et al., 2012). cn.MOPS is a multiple sample read depth method that applies a Bayesian approach to decompose read variations across multiple samples into integer copy numbers and noise by its mixture components and Poisson distributions, respectively. cn.MOPS avoids read count biases along the chromosomes by modeling the depth of coverage across all samples at each genomic position. The cn.MOPS program was run using a window length of 1 kb, mean normalization mode, and the default values for all other parameters. Autosomal and sex chromosomes were processed differently due to differences in expected ploidy of the genome. Autosomal CNVs, which are expected to be diploid, were identified using all 240 samples. CNVs on the sex chromosomes were identified by processing the 167 males and 73 females separately, as SSCX is expected to be diploid in female samples and SSCX and SSCY are expected to be haploid in the male samples.

CNVs identified by LUMPY that were at least 10% overlapped by a CNV identified by cn.MOPS, meaning that the ratio of the number of bp overlapped between the LUMPY CNV and at least one cn.MOPs CNV to the length of the LUMPY CNV was greater than 0.10, were retained for downstream analysis. Next, CNVs were used to construct a set of copy number variable regions (CNVRs). A CNVR was constructed by merging CNVs across samples that exhibited at least 50% pairwise reciprocal overlap in their genomic coordinates. For example, suppose we have two CNVs, CNV1 beginning at position *a* and ending at position *b* and CNV2 running from *c* to *d* with *a* < *c* < *b* < *d*. If the reciprocal overlap between the two CNVs is at least 50%, then they are merged into a CNVR that runs from *a* to *d* on the genome.

### Validation of CNVR Using Data From Sequenced Parent–Offspring Trios

For the transmission rate (paternal and maternal), in each parent–child pair, CNVRs in the parent also called in the child were counted and then divided by the total number of CNVR calls in the parent. For the inheritance rate, CNVR calls in the child also present in at least one parent were counted and then divided by the total number of CNVRs in the child.

### Gene Content and GO

Genes from the NCBI *Sus scrofa* annotation (Release 106) overlapping by at least 1 bp with CNVRs were identified. Functions of protein-coding CNV-overlapped genes were determined using the PANTHER classification system (Version 14.0, Mi et al., 2013).

Enrichment analysis of gene function was performed using PANTHER’s implementation of the binomial test of overrepresentation. Significance of GO terms was assessed using the default *Sus scrofa* GO annotation as the reference set for the enrichment analysis, and data were considered statistically significant at a Benjamini-Hochberg-corrected *P* value < 0.05.

### Enrichment of Quantitative Trait Loci

Enrichment analysis of quantitative trait loci (QTL) overlapped with CNVR was performed using Fisher’s exact test. Data were considered statistically significant at a Benjamini-Hochberg-corrected *P* value < 0.05.

## Results and Discussion

### Sequencing and Read Mapping

Genomic DNA from 240 pigs, from a composite population at USMARC, was sequenced on Illumina HiSeq and NextSeq platforms, generating approximately 72 billion paired-end reads (**Table S1**). Sequence reads covered each pig’s genome at a mean of 13.62-fold (×) coverage. Individual coverage per animal ranged from 0.97× to 31.13×; 24 animals were covered at less than 3×, and 44 were covered at more than 20×.

When generating our sequence data, we targeted a minimum of 3× coverage for each of the founding sows and 10× coverage for the remaining 168 animals. However, there was considerable variation around the 3.66× and 18.41× mean coverage for the founding sows and other animals, respectively. Some of this variation can be attributed to technical aspects of NGS technology, such as the stochasticity of sequencing, DNA quality, and library preparation. The combined sequence from all 240 animals covered 99.99% of the reference genome.

### CNVR Discovery and Statistics

CNVs in the genome of the 240 pigs were identified by taking the overlap of two methods: (1) a combination of CNVnator and LUMPY and (2) cn.MOPS. Most of the previous NGS CNV studies in swine have utilized read depth approaches to identify variants (Paudel et al., 2013; Jiang et al., 2014; Paudel et al., 2015; Wang H. et al., 2015; Wang et al., 2016; Revilla et al., 2017). Although read depth can be a powerful tool to identify CNV, often the boundaries are not well determined because of the sliding window approach. The exact boundaries of CNV events can be important for determining their functional effect (e.g., affecting coding sequence). Other CNV detection strategies, such as paired-end mapping or split reads, can be used to fine map CNV and determine more precise boundaries of the variants. The CNVnator–LUMPY combination approach used in this work calls CNV in individual samples utilizing paired-end mapping, split reads, and read depth. Although this method should give more accurate CNV breakpoints than read depth signal alone, single sample CNV callers are known to suffer from decreased detection power and high false-positive rates. A total of 2,079,579 were identified using CNVnator–LUMPY. Utilization of data from multiple samples has been shown to improve CNV detection (Klambauer et al., 2012; Duan et al., 2014). Therefore, as a further error correction step, CNVs were also detected using a multiple sample read depth caller, cn.MOPS (695,741 CNV identified). A total of 39,315 CNVs, overlapping between the two methods, were retained for downstream analysis (**Table S2**). CNVs were merged across each genome and then across samples into CNVRs, and CNVRs less than 200 bp in length were filtered out. This resulted in a final set consisting of 3,538 CNVRs (**Table S3**), including 1,820 novel CNVRs that were not reported in previous studies.

Note that approximately 19% (45 of 240) of the animals in this study had low to moderate sequence depth (<5× coverage). The highest sensitivity and resolution in CNV detection are attained through high coverage sequencing (>10×; Alkan et al., 2011). However, until sequencing costs drop dramatically, it is not feasible, in most cases, to generate high coverage genomic sequence on large numbers of animals. We consider low-coverage sequencing data here, because methods for analyzing SNP and CNV in low-coverage data will continue to be relevant in the future in terms of a study’s discovery power, where a fixed number of reads should rather be used for sequencing more samples with lower coverage than for sequencing fewer samples with higher coverage (Le and Durbin, 2011). Due to the cost-effectiveness of sequencing at lower coverage, recent studies have explored strategies for using low-coverage sequence to detect common CNV that could explain a significant amount of phenotypic variation (Keel et al., 2016a; Zhou et al., 2018). Both CNVnator and cn.MOPS have been shown to have moderate to high accuracy in detecting CNV from low-coverage sequence in diploid genomes (Keel et al., 2016a; Malekpour et al., 2018), particularly in data sets consisting of samples with mixed levels of coverage. Therefore, the use of these methods, coupled with LUMPY, should provide reasonably accurate results for CNV calling in our 240 animals.

Sizes of the CNVRs ranged from 0.203 to 398.9 kb, with an average of 6.8 kb and a median of 2.9 kb. The CNVR occupied a total of 22.9 unique Mb or 0.94% of the *Sus scrofa* genome. The CNVR coverage of the genome is lower than the results of previous reports in swine (4.0%; Jiang et al., 2014) and other species, including mouse (6.87%; Locke et al., 2015), human (12%; Redon et al., 2006), and cattle (6.7%; Keel et al., 2016a), which may be due to our stringent criteria (e.g., requiring detection with two different approaches). Among the CNVRs, 144 showed copy number gain (duplication), 3372 showed copy number loss (deletion), and 22 showed a mix of copy number loss and gain from different individuals. Clearly, there was a large discrepancy in the numbers of duplication and deletion CNVR. Overall, read-depth methods are more sensitive to deletion CNV calls than duplication calls, especially in mid- to low-coverage sequence data, as it is easier to identify a “missing” segment of the genome than an amplified one with limited sequence reads. In fact, 3.1% (105 of 3372) of deletion calls were identified in only animals with <10× coverage. As low-coverage WGS continues to become more widely utilized, it will be necessary to focus on adapting CNV calling tools to this type of data.

### Distribution of CNVR

**Figure 1:**
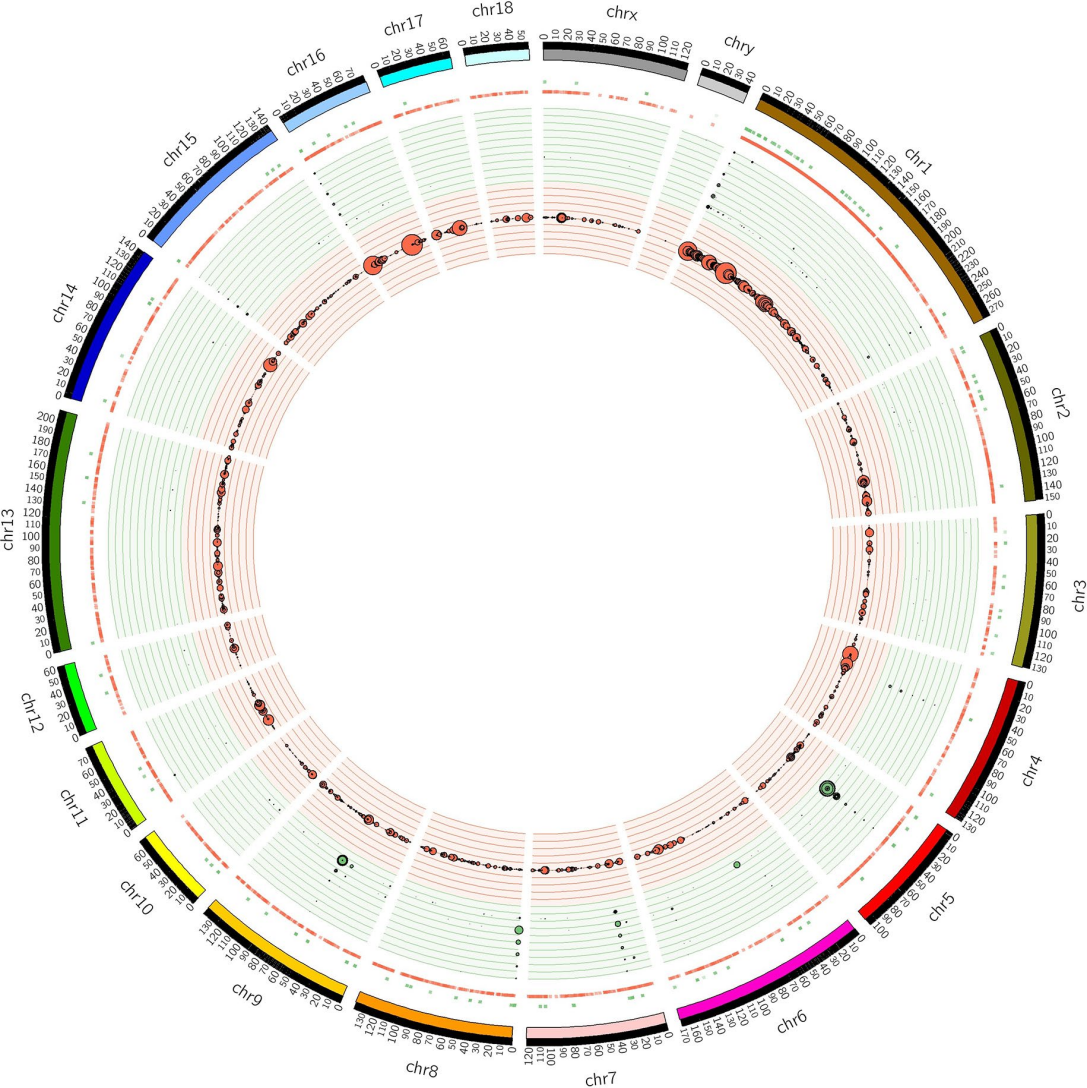
Positions of CNVRs identified from the 240 sequenced swine genomes in Circos format (Krzywinski et al., 2009). The outer ideogram runs clockwise from chromosome 1 to chromosome Y with labels in Mb of physical distance. The copy number data are represented in the inner tracks. The two innermost tracks show scatter plots of the CNVR, where the red track shows copy number loss and the green track shows copy number gain. Concentric circles within these tracks indicate *y*-axis values in the scatter plot. The 10 concentric circles in the red track mark values 0 ≤ *y* < 2, with 0 being the innermost track, while the 11 concentric circles in the green track mark values 2 ≤ *y* ≤ 8. The size of the dot in the scatter plot is proportional to the number of samples containing the CNVR. The other track shows a heat map that indicates the parts of the genome that contain copy number gain and loss. This plot simply collapses the scatter plot values onto a single radial position.

The distribution of CNVRs along each of the chromosomes is shown in **Figure 1**. Variants were not uniformly distributed on the chromosomes. The number of CNVRs was strongly correlated with the size of the chromosome (Pearson correlation coefficient *r* = 0.77). SSC1 and SSC13 exhibited the largest numbers of CNVRs (1231 and 231, respectively), while SSCY, SSC18, and SSC12 had the smallest numbers (2, 49, and 52 CNVRs, respectively). On average, 0.79% of each chromosome was covered by CNVRs (**Table 1**).

**Table 1:** CNVR distribution across the genome.

Chromosome	Chromosome length (bp)	No. of CNVR on chromosome	Unique bp covered by CNVR	Ratio covered by CNVR
SSC1	274330532	1231	7969003	2.90%
SSC2	151935994	192	1318835	0.87%
SSC3	132848913	101	570813	0.43%
SSC4	130910915	137	523004	0.40%
SSC5	104526007	146	1132811	1.08%
SSC6	170843587	152	772899	0.45%
SSC7	121844099	141	638302	0.52%
SSC8	138966237	203	1599178	1.15%
SSC9	139512083	167	1088850	0.78%
SSC10	69359453	82	407652	0.59%
SSC11	79169978	103	1338230	1.69%
SSC12	61602749	52	337802	0.55%
SSC13	208334590	231	1636688	0.79%
SSC14	141755446	126	1099698	0.78%
SSC15	140412725	141	734861	0.52%
SSC16	79944280	127	449040	0.56%
SSC17	63494081	62	277525	0.44%
SSC18	55982971	49	433245	0.77%
SSCX	125939595	93	582165	0.46%
SSCY	43547828	2	17943	0.04%

The number of CNVRs per animal ranged from 0 to 348, with a mean of 157.8 (**Table S4**). CNVs spanned up to 0.13% of the genome of each animal, with a mean and median of 0.057% and 0.062%, respectively. This variation across individuals can be partially explained by differences in genomic sequencing coverage. Smaller numbers of CNVRs were identified in samples with low sequencing depth, and the number of identified CNVRs tended to increase as genomic coverage increased (Pearson correlation coefficient *r* = 0.84).

**Figure 2:**
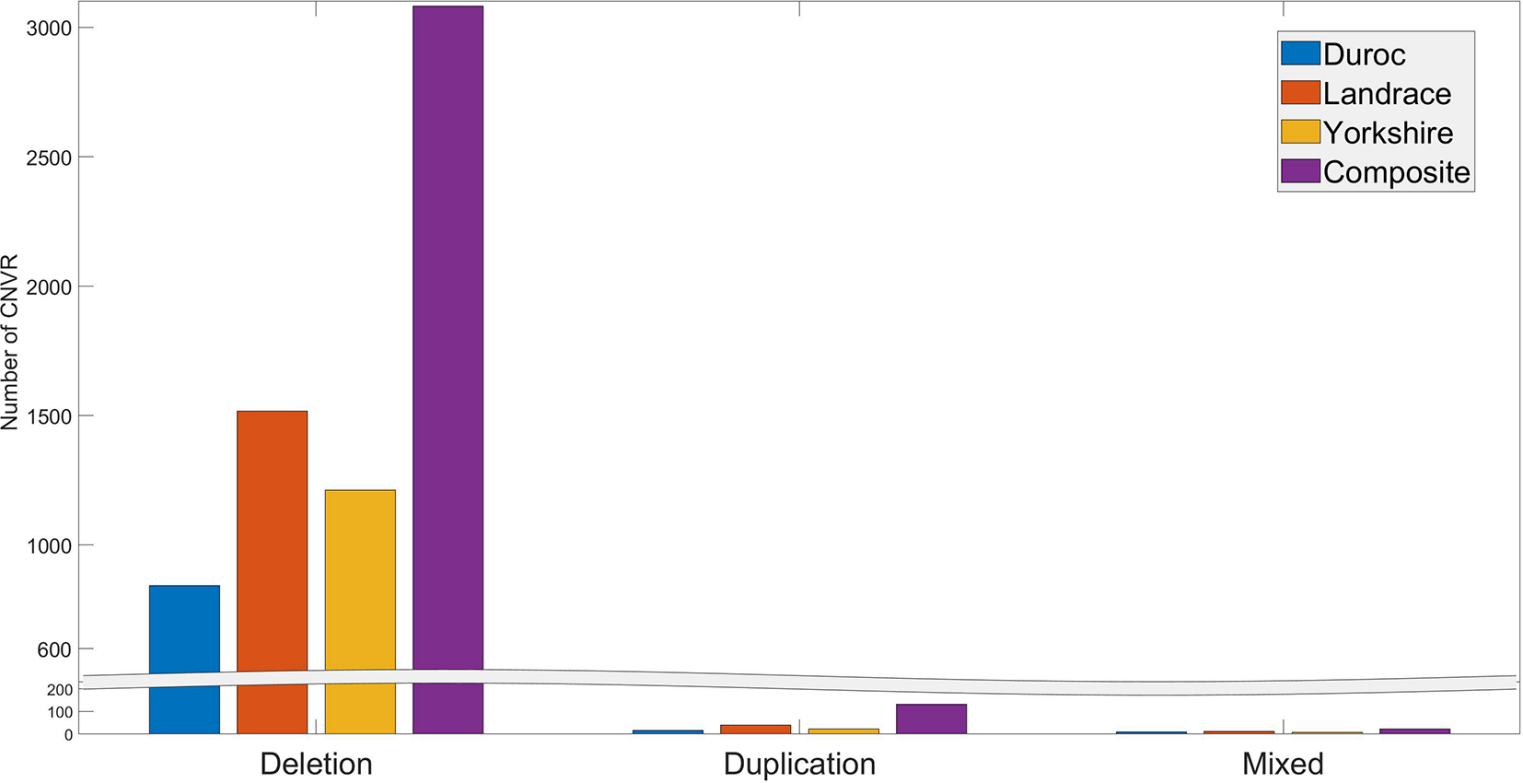
Distribution of CNVR types across breeds.

The number of individuals exhibiting each CNVR ranged from 1 to 175. Many CNVRs (∼2649) were present in a small percentage (< 5%) of the animals. Three CNVRs (CNVR 2103, 1676, and 2104 in **Table S3**) were present in more than 60% of the population. The distribution of deletion, duplication, and mixed CNVRs across breeds is shown in **Figure 2**. The purebred Landrace and Yorkshire boars and the composite animals had more CNVR of all three types than the purebred Duroc boars. This is likely because the Sscrofa 11.1 reference genome assembly was obtained from a Duroc animal.

A total of 1620 CNVRs were found to be breed-specific in origin (**Table S5**). Most (64%) breed-specific CNVRs were present in only one animal. Breed-specific CNVRs that were present in the largest numbers of animals were found in the composite breed. This is likely due to the larger number of composite animals in the data set. Increased numbers of animals in the three pure breeds will be necessary to conduct a complete breed-of-origin analysis. This will be a focus of future work.

### CNVR Concordance in Parent–Offspring Trios

Pedigree data from 12 sequenced parent–offspring trios were used as a substitute for molecular validation, which we have chosen to forego since this work was intended to be a discovery. In a follow-up study, we are planning to look for CNVRs that may associate with phenotypes in our population and validation using PCR methods will be performed for those CNVRs. If a CNV is transmitted from parent to offspring, then it can be considered validated. Although this type of validation is not 100% accurate, it is satisfactory to allow us to estimate error rates. In an ideal data set, paternal and maternal transmission rates would be 50%, and inheritance rates would be 100%. Deviations from this ideal could be explained by multiple factors. Both false-positive and false-negative CNV calls will cause a decrease in transmission and inheritance rates. Another possible factor is *de novo* mutations in offspring, which will not affect transmission rates, but will affect inheritance rates. Additionally, there is the possibility of somatic mutation in one or more of the parents, essentially a *de novo* mutation in parents as they age. Somatic mutations would affect transmission rates but not inheritance rates. All of these factors could potentially affect the data simultaneously. Therefore, they cannot be individually estimated. However, assuming that *de novo* and somatic mutations are rare compared to CNVR calling errors, we can use transmission and inheritance rates to estimate error rates.

**Table 2** shows the paternal and maternal transmission rates and the inheritance rate for each of the 12 sets of trios. The average transmission rates were 37.7% and 41.4% for maternal and paternal, respectively. These rates are much closer to the ideal 50% transmission rate than what was reported in a similar study in humans (27% for maternal and 28% for paternal; Zheng et al., 2012). The average inheritance rate was 52%, which falls between inheritance rates reported in previous studies, 42% in Zheng et al. (2012) and 74.8% in Wang et al. (2007). Therefore, under the assumption that the *de novo* and somatic mutations are rare, we approximate the error rate in CNVR calls to be 48% (100% minus the inheritance rate). This error rate is comparable to previously published results for several different CNV-calling algorithms for whole-genome sequence data (26%–77%; Legault et al., 2015). This consistency suggests that high error rates may be due to algorithmic issues rather than the input data. Clearly, further development of bioinformatics protocols and tools for producing high-confidence, consistent CNV calls is necessary to improve the quality of CNV discovery studies.

**Table 2:** Transmission and inheritance rates in parent–offspring trios.

Trio number	Paternal transmission rate	Maternal transmission rate	Inheritance rate
1	0.277	0.270	0.549
2	0.414	0.352	0.453
3	0.351	0.519	0.524
4	0.370	0.369	0.356
5	0.396	0.714	0.417
6	0.373	0.448	0.576
7	0.393	0.382	0.570
8	0.428	0.403	0.482
9	0.428	0.367	0.603
10	0.401	0.390	0.441
11	0.360	0.408	0.668
12	0.335	0.343	0.594
Mean	0.377	0.414	0.520

### Comparison of CNVRs with Previous Studies

Comparison of our results with CNVRs identified in several previous swine studies showed varying levels of overlapping CNVRs between studies (**Table 3**). Here, we used a much less stringent definition of overlap than that used in identifying overlapping CNV, where two CNVRs were considered overlapped as long as they shared at least one base.

**Table 3:** Comparison of CNVRs identified in this study to results from other studies (based on the Sscrofa 11.1 genome assembly).

Platform	Findings from other studies				CNVR overlap with this study	
	Study^a^	Breeds	Samples	No. of CNVRs (No. before mapping)	No. of overlapped CNVRs from this study	Ratio of overlapped CNVRs from this study
CGH-based study	Fadista et al., 2008**	1	12	31 (37)	1	0.00%
	Li et al., 2012*	8	12	241 (259)	47	1.33%
	Wang et al., 2014**	9	12	48 (52)	8	0.22%
	Wang J. et al., 2015**	9	12	602 (689)	129	3.65%
SNP-based study (PorcineSNP60)	Ramayo-Caldas et al., 2010*	2	55	47 (49)	104	2.94%
	Chen et al., 2012**	18	1693	537 (565)	262	7.41%
	Wang et al., 2012*	3	474	357 (382)	130	3.67%
	Wang L. et al., 2013**	2	585	234 (249)	670	18.94%
	Schiavo et al., 2014**	1	305	166 (170)	100	2.83%
	Wiedmann et al., 2015**	3	1802	480 (502)	579	16.37%
	Long et al., 2016**	3	905	3746 (6193)	182	5.14%
	Xie et al., 2016**	2	120	166 (172)	84	2.37%
	Hay et al., 2017**	1	660	256 (271)	189	5.34%
SNP-based study (Infimum II)	Wang J. et al., 2013**	10	14	57 (63)	20	0.57%
Next-Generation Sequencing	Paudel et al., 2013**	13	16	2880 (3118)	70	1.98%
	Jiang et al., 2014**	10	13	2820 (3131)	238	6.73%
	Paudel et al., 2015**	5	16	1238 (1408)	33	0.93%
	Wang H. et al., 2015**	13	49	2359 (3131)	53	1.50%
	Wang et al., 2016**	6	252	433 (455)	13	0.37%
	Revilla et al., 2017**	2	7	508 (540)	153	4.32%
This Study		3	240	3538		

^a^Original data set was mapped to Sscrofa 9.2 or Sscrofa 10.2 assembly, denoted by * and **, respectively. CNVRs were converted to Sscrofa 11.1 coordinates using the NCBI Genome Remapping tool. Successfully mapped CNVRs are shown in the CNVR column with the original number of published CNVRs shown in parentheses.

Generally speaking, percentages of overlap in CNV events identified between this work and previous studies were low (average of 4.33% overlap). This result is very similar to what has been observed in cattle CNV studies, where typically <40% overlap exists between studies (Keel et al. 2016b). These discrepancies are likely driven by many technical aspects of the experiments, including vastly different sample sizes, differences in breeds and the number of breeds represented, detection platform (array-based vs. NGS), and CNV detection algorithms. Many of the CNV discovery studies in swine have involved pure and half Chinese breeds. Therefore, it is likely that many of the CNVRs identified in those studies do not segregate in our population. Our population represents commercial swine germplasm and, because of domestication and selection for lean meat production and reproductive efficiency, has diverged from germplasm studied in other experiments.

It should be noted that two of the three studies with highest overlap percentages, Wang et al. (2013) and Wiedmann et al. (2015), were those that had high representations of Yorkshire, Landrace, and Duroc animals. In fact, the study of Wiedmann et al. (2015) was performed on animals from the same population used in this study. The discrepancy in CNV identified in their study and ours is likely due to differences in platform (SNP beadchip vs. whole-genome sequence), detection algorithm, and genome build (Sscrofa 10.2 vs. Sscrofa 11.1).

### Function of CNV-Overlapped Genes

A total of 1401 genes from the NCBI annotation of the Sscrofa 11.1 genome were identified to be overlapping with our detected CNVRs (**Table S3**), including 911 protein-coding genes, 58 pseudogenes, 273 non-coding RNA, and 160 miscellaneous RNA. CNV-overlapped genes were overlapped with 2314 CNVRs. Using PANTHER’s functional annotation tool to inspect GO slim terms mapping to protein-coding CNV-overlapped genes, we identified that many of these genes were involved in binding (34.7%), catalytic activity (35.7%), metabolic process (23.1%), biological regulation (20.3%), cellular process (11.4%), localization (9.3%), and molecular transducer activity (9.2%).

Enrichment analysis was performed, using the *Sus scrofa* GO database to identify GO terms that were significantly enriched in our gene set. GO enrichment analysis showed that biological process terms related to regulation of ion transport, cell adhesion, signaling, nervous system development, neurogenesis, and locomotion, as well as molecular function terms related to glutamate receptor activity, protein binding, enzyme binding, ATP binding, and neurotransmitter receptor activity, were significantly overrepresented in the genes overlapped by CNVR (Benjamini-Hochberg-corrected *P* value <0.05; **Table S6**).

Approximately 3.6% of the CNV-overlapped genes belonged to the OR gene family, one of the largest gene families in the porcine genome (Groenen et al., 2012; Nguyen et al., 2012). ORs are G-protein-coupled receptors involved in signal transduction and have been found to be copy number variable in many mammalian species, including human (Young et al., 2008), rat (Guryev et al., 2008), mouse (Pezer et al., 2015), swine (Chen et al., 2012; Wang et al., 2012; Paudel et al., 2013; Wang J. et al., 2013; Paudel et al., 2015), and cattle (Liu et al., 2010; Keel et al., 2016b; Xu et al., 2016). Young et al. (2008) showed that OR genes displayed varying copy numbers among 50 people, and that this variation may play a role in olfactory ability and sensitivity. It is also thought that ORs may play a chemosensory role as they are expressed on sperm and thought to direct them to the egg *via* chemotaxis (Spehr et al., 2006). Paudel et al. (2015) identified that OR genes were overrepresented among CNVRs across several members of the *Sus* genus. These genes may have been important components of swine evolution, as scent would have been critical for foraging for food, avoiding predators, and finding a mate.

### Overlap and Enrichment of Known QTL in CNVRs

To reveal the potential relationships between CNVRs and QTL, we analyzed the overlap between our CNVRs and known swine QTL and performed QTL enrichment analyses. Swine QTL from the Sscrofa 11.1 genome build were downloaded from the Animal QTL database (Release 34; http://www.animalgenome.org/cgi-bin/QTLdb/SS/index), which includes 26,076 known QTL for 647 different traits. QTL overlapping with CNVRs were identified (**Table S7A**), traits were ranked according to the number of QTL/CNVR overlaps (**Table S7B**), and QTL enrichment analysis was performed (**Table S7B**). The 10 highest ranked traits included drip loss (519 overlaps), average daily gain (235 overlaps), average backfat thickness (195 overlaps), loin muscle area (179 overlaps), backfat at last rib (153 overlaps), teat number (127 overlaps), carcass length (95 overlaps), ham weight (81 overlaps), backfat at tenth rib (75 overlaps), and lean meat percentage (73 overlaps).

QTL enrichment analysis, using QTL from the Animal QTL database overlapping with CNVR (*n* = 525 traits), identified that QTL for 132 traits were significantly enriched. The most significantly enriched QTL was drip loss (*P* = 4.09E−99). Several meat quality traits, including average back fat thickness, loin muscle area, ham weight, carcass weight, and dressing percentage, were also found to be among the most significantly enriched.

Approximately 840 QTL have been previously reported from GWAS utilizing animals from the same experimental herd used in this study (**Table 4**). QTL/CNVR overlaps were identified (**Table S8A**), traits were ranked according to the number of overlaps (**Table S8B**), and QTL enrichment analysis was performed (**Table S8B**). The highest ranked traits included vertebra number (28 overlaps), as well as several reproductive traits including age of puberty (41 overlaps), ovulation rate (18 overlaps), % stillborn ignoring the last piglet (18 overlaps), and last birth interval (17 overlaps). It should be noted that, in this work, CNVRs were not tested for statistical association with QTL, but rather the overlapping genomic positions of the latter were used as one indicator of the potential function of the CNVRs.

**Table 4:** QTL identified in USMARC swine population from previously published GWAS.

Trait	Number of reported QTL (number overlapping with CNVR)	Publication
Age of puberty	222 (41)	Nonneman et al., 2016
Litter average birth interval (minus last birth)	25 (15)	Schneider et al., 2015
Last birth interval	25 (17)	Schneider et al., 2015
Number stillborn (ignoring last piglet)	25 (13)	Schneider et al., 2015
Number stillborn in last birth position	25 (16)	Schneider et al., 2015
Percent stillborn (ignoring last piglet)	25 (18)	Schneider et al., 2015
Total number born	11 (2)	Schneider et al., 2012
Number born alive	14 (4)	Schneider et al., 2012
Number born dead	1 (0)	Schneider et al., 2012
Litter birth weight	33 (5)	Schneider et al., 2012
Average piglet birth weight	65 (9)	Schneider et al., 2012
Temperature humidity feeding behavior comparison normal-alert temperatures	17 (6)	Cross et al., 2018
Temperature humidity feeding behavior comparison normal-danger temperatures	13 (3)	Cross et al., 2018
Temperature humidity feeding behavior comparison normal-emergency temperatures	13 (6)	Cross et al., 2018
Temperature humidity feeding behavior comparison alert-danger temperatures	13 (4)	Cross et al., 2018
Temperature humidity feeding behavior comparison alert-emergency temperatures	6 (3)	Cross et al., 2018
Temperature humidity feeding behavior comparison danger-emergency temperatures	4 (2)	Cross et al., 2018
Immunocrit	36 (0)	Rohrer et al., 2014
Shear force	3 (0)	Nonneman et al., 2013
Intramuscular fat	31 (0)	Nonneman et al., 2013
Minolta color score L*	3 (0)	Nonneman et al., 2013
Minolta color score b*	2 (0)	Nonneman et al., 2013
Cookloss	11 (0)	Nonneman et al., 2013
pH	10 (0)	Nonneman et al., 2013
Purge	8 (0)	Nonneman et al., 2013
Ovulation rate	96 (18)	Schneider et al., 2014
Teat number	36 (0)	Rohrer and Nonneman, 2017
Vertebra number	49 (28)	Rohrer et al., 2015
Kyphosis	16 (11)	Lindholm-Perry et al., 2010

Of the 20 GWAS traits that had QTL overlapping with CNVR, 7 of them were found to be significantly enriched. These included vertebra number (*P* = 4.35E−07), percent stillborn ignoring the last piglet (*P* = 4.64E−07), last birth interval (*P* = 2.79E−06), number stillborn in the last birth position (*P* = 1.58E−05), litter average birth interval minus the last birth (*P* = 8.13E−05), kyphosis (*P* = 1.49E−05), and number stillborn ignoring the last piglet (*P* = 1.49E−04).

These results are similar to those from a study conducted by Revay et al. (2015), where age of puberty and teat number were found to be the most abundant reproductive QTL overlapped by swine CNVRs. This coupled with the overrepresentation of GO terms such as cell motility, nervous system development, and organ development in CNVR-overlapped genes suggests that CNVR may play a role in shaping various reproductive traits in swine.

## Conclusion

CNV continues to gain considerable interest as a source of genetic variation that may play a role in phenotypic diversity. Swine CNV research has made significant progress in the last 5 years. Genome-wide surveys of CNV have been conducted using a variety of platforms and algorithms. Studies that utilize NGS data have been limited in swine. Moreover, much of the NGS-based studies have focused on diverse pig breeds and wild boars from different regions of the world rather than commercial breeds. To capture CNV present in commercial swine germplasm, we utilized whole-genome sequence from 240 animals. Our study is one of the largest sequence-based swine CNV studies to date.

We identified 1401 genes overlapping with CNVRs. GO enrichment analysis showed that our set of CNV-overlapped genes was enriched with genes involved in organism development, and QTL analysis showed that CNVRs overlapped with many QTL for reproductive traits. These results are consistent with findings in other swine CNV studies, which suggests that CNV may play a role in shaping reproductive traits. Understanding the exact role that CNV plays in reshaping gene structure, modulating gene expression, and ultimately contributing to phenotypic variation are open questions. The focus of our future work will be to develop strategies for CNV imputation, identify CNVs that associate with phenotypes in our population, and validate those CNVs using methods such as digital droplet PCR, with the long-term goal of discovering the extent to which CNVs affect economic traits of interest and developing strategies for incorporating them into genomic selection systems.

## Data Availability

The raw data supporting the conclusions of this manuscript will be made available by the authors, without undue reservation, to any qualified researcher.

## Disclaimer

Mention of trade names or commercial products in this publication is solely for the purpose of providing specific information and does not imply recommendation or endorsement by the U.S. Department of Agriculture.

The U.S. Department of Agriculture (USDA) prohibits discrimination in all its programs and activities on the basis of race, color, national origin, age, disability, and where applicable, sex, marital status, familial status, parental status, religion, sexual orientation, genetic information, political beliefs, reprisal, or because all or part of an individual’s income is derived from any public assistance program. (Not all prohibited bases apply to all programs.) Persons with disabilities who require alternative means for communication of program information (Braille, large print, audiotape, etc.) should contact USDA’s TARGET Center at (202) 720-2600 (voice and TDD). To file a complaint of discrimination, write to USDA, Director, Office of Civil Rights, 1400 Independence Avenue, S.W., Washington, D.C. 20250-9410, or call (800) 795-3272 (voice) or (202) 720-6382 (TDD). USDA is an equal opportunity provider and employer.

## Author Contributions

BK conceived the study, and BK, DN, and GR participated in its design and coordination. DN, GR, WO, and AL-P were involved in the acquisition of data, and BK performed all data analysis. BK drafted the manuscript, and DN, GR, WO, and AL-P contributed to the writing and editing. All authors read and approved the final manuscript.

## Conflict of Interest Statement

The authors declare that the research was conducted in the absence of any commercial or financial relationships that could be construed as a potential conflict of interest.

## Supplementary Material

Table S1 Sequencing statistics.

Table S2 CNV overlapping between Lumpy and cn.MOPS analyses.

Table S3 CNVR and their overlapping genes.

Table S4 Distribution of CNVR in individual animals.

Table S5 Breed specific CNVR.

Table S6 Results from GO enrichment analysis.

Table S7 Results from QTL enrichment analysis using QTL from the AnimalQTL Database.

Table S8 Results from QTL enrichment analysis using previously identified QTL in the USMARC swine population.
